# CDC Grand Rounds: Global Tobacco Control

**Published:** 2014-04-04

**Authors:** Samira Asma, Yang Song, Joanna Cohen, Michael Eriksen, Terry Pechacek, Nicole Cohen, John Iskander

**Affiliations:** 1 Office on Smoking and Health, National Center for Chronic Disease Prevention and Health Promotion, CDC; 2 Johns Hopkins Bloomberg School of Public Health, Baltimore, Maryland; 3 Georgia State University School of Public Health, Atlanta, Georgia; 4 Office of the Director, CDC

During the 20th century, use of tobacco products contributed to the deaths of 100 million persons worldwide (1). In 2011, approximately 6 million additional deaths were linked to tobacco use, the world’s leading underlying cause of death, responsible for more deaths each year than human immunodeficiency virus/acquired immunodeficiency syndrome (HIV/AIDS), tuberculosis, and malaria combined (1). One third to one half of lifetime users die from tobacco products, and smokers die an average of 14 years earlier than nonsmokers (2,3). Manufactured cigarettes account for 96% of all tobacco sales worldwide. From 1880 to 2009, annual global consumption of cigarettes increased from an estimated 10 billion cigarettes to approximately 5.9 trillion cigarettes (Figure 1), with five countries accounting for 58% of the total consumption: China (38%), Russia (7%), the United States (5%), Indonesia (4%), and Japan (4%). Among the estimated 1 billion smokers worldwide, men outnumber women by four to one. In 14 countries, at least 50% of men smoke, whereas in more than half of these same countries, fewer than 10% of women smoke (4). If current trends persist, an estimated 500 million persons alive today will die from use of tobacco products. By 2030, tobacco use will result in the deaths of approximately 8 million persons worldwide each year (4). Yet, every death from tobacco products is preventable.

## The Tobacco Industry

Tobacco plants are grown in 124 countries. China, which produces 43% of the world’s tobacco, has seen a 200% increase in production over the past 30 years. Other leading producers include Brazil, India, the United States, Argentina, Malawi, and Indonesia. There are five major, private tobacco companies throughout the world and 16 state-owned companies. The largest state-owned company, China National Tobacco Corporation, produces one third of the cigarettes sold worldwide. In 2010, the combined total revenue of the top six tobacco companies in the world was approximately $346 billion with a combined profit of $35 billion (4). In the United States, marketing expenditures for cigarette advertising and promotion reached $9.9 billion in 2008; 83% of this total was spent on price discounts, coupons, and retail value–added promotions (5).

## Global Public Health Interventions and Proven Strategies to Reduce Tobacco Use

In 2005, the World Health Organization’s Framework Convention on Tobacco Control (WHO FCTC) was codified as the world’s first international public health treaty. Ratified by 178 parties, WHO FCTC calls for global, coordinated actions aimed at reducing tobacco use (6).

**FIGURE 2:**
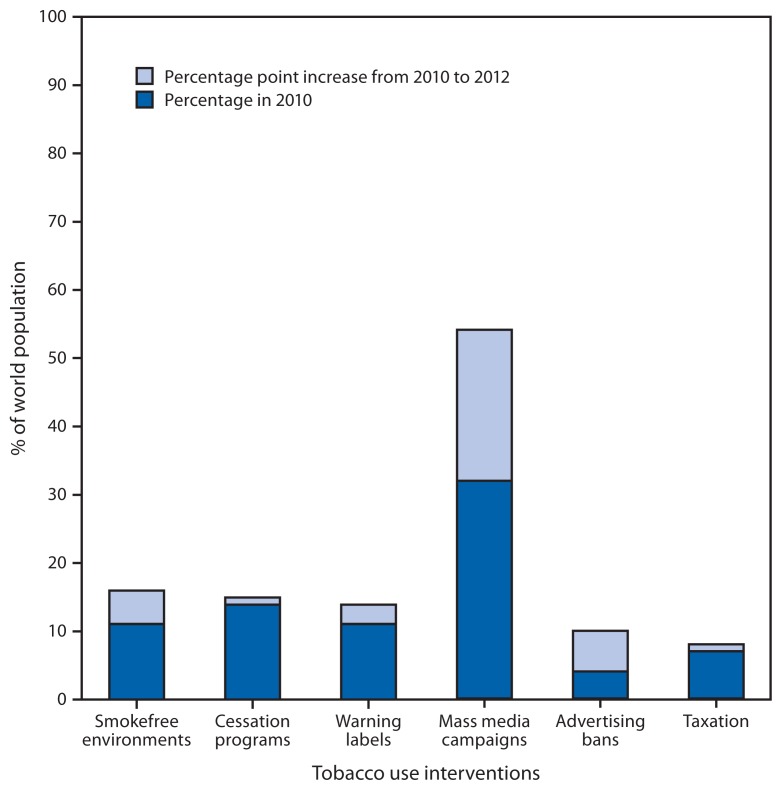
Percentage of the world population covered by MPOWER interventions against tobacco use — 2010 and 2012 **Source:** World Health Organization. WHO report on the global tobacco epidemic 2013: enforcing bans on tobacco advertising, promotion, and sponsorship. Geneva, Switzerland: World Health Organization; 2013. Available at http://www.who.int/tobacco/global_report/2013/en.

In 2008, WHO introduced its MPOWER measures as practical, cost-effective ways to scale up global implementation of specific WHO FCTC provisions. The six measures of MPOWER are 1) monitoring tobacco use and prevention programs and policies; 2) protecting persons from secondhand smoke through establishment of smokefree public places; 3) offering persons help to quit tobacco use; 4) warning about the dangers of tobacco use through mass media campaigns and labels on tobacco packages; 5) enforcing bans on tobacco advertising, promotion, and sponsorship; and 6) raising taxes on tobacco products (2) (Figure 2).

CDC has focused much of its global contribution to MPOWER on monitoring and surveillance through the Global Tobacco Surveillance System (GTSS), a set of globally standardized surveys designed to monitor tobacco use as well as progress in tobacco control policy measures.* GTSS enhances countries’ capacity to design, implement, monitor, and evaluate tobacco control policies.

### Monitoring tobacco use and control

GTSS includes surveys designed for youths (the Global Youth Tobacco Survey [GYTS]) and adults (the Global Adult Tobacco Survey [GATS]), as well as Tobacco Questions for Surveys (TQS). GYTS is a school-based survey of students aged 13–15 years that uses a standard protocol. Since 1999, GYTS has been conducted in approximately 180 countries. Many countries have conducted the survey multiple times, providing comparable results within and among countries over time. Key GYTS results include the finding that 10% of students aged 13–15 years currently smoke cigarettes, and 10% use other tobacco products. Additionally, 25% of smokers in this age group first tried cigarettes by the age of 10 years, and two thirds want to quit. Approximately 40% of students are exposed to secondhand smoke in the home, and 50% are exposed in public places (7).

GATS is a nationally representative, household survey of adults (aged ≥15 years) that is used to track tobacco use and evaluate tobacco control policies. Since 2008, it has been completed in 22 countries, covering 61% of the world’s adult population and 63% of the world’s smokers. Findings from the 19 GATS countries with publicly available data indicate that approximately 875 million adults currently use tobacco, although 19% of smokers plan to or are thinking about quitting. At least 391 million adults are exposed to secondhand smoke at the workplace, and 15% of adults noticed cigarette marketing in stores where cigarettes are sold (8).

TQS contains a list of 22 survey questions that can be integrated into national, subnational, and international surveys to promote data comparability within and across countries over time. It has been implemented in 20 countries.

### Smokefree laws and regulations

Comprehensive and well-enforced smokefree policies result in changes in social norms and attitudes toward smoking, with concomitant decreases in cigarette consumption and increases in quitting (9). The number of smokefree areas in the United States and around the world doubled from 2008 to 2010. Besides being a simple and low-cost way to protect populations from exposure to secondhand smoke, smokefree laws receive strong public support, which typically increases after the policies go into effect, even among smokers, and do not harm businesses (9,10).

### Cessation programs

The majority of smokers quit without assistance (11); however, cessation interventions can greatly increase quit rates. Persons who discontinue tobacco use receive immediate and significant health benefits and have most of their excess health risks reduced within a few years. GATS results from 19 countries show that the two countries with the highest proportions of persons who have quit smoking are Brazil and Uruguay, both of which have implemented comprehensive tobacco control programs, including cost-covered cessation services (2,8).

### Warning labels

Requiring graphic warning labels on cigarette packages is another effective tobacco control strategy (12). Warning labels should be large with dramatic images, include specific health warnings, and should be changed periodically. In Brazil, where graphic warning labels have been required since 2002 (13), more than 70% of smokers approved of these labels, with over half of those surveyed reporting that they had changed their opinions about the health consequences of smoking, and nearly 70% of smokers stating that they wanted to quit as a result of the labels (1). To further limit the attractiveness and appeal of cigarette packages, in December 2012 Australia became the first country to adopt plain, standard packaging that eliminates all color, imagery, and brand appeal (2).

### Tobacco advertising, promotion, and sponsorship bans

Comprehensive tobacco marketing bans that regulate advertising, promotion, and sponsorship can also reduce tobacco’s appeal (2). Comprehensive bans have been shown to reduce average cigarette consumption by 9% within the 10 years after implementation, compared with just 1% in countries without such bans (14). In addition, aggressive antismoking media campaigns (sometimes conducted in conjunction with providing access to cessation services) prevent tobacco use initiation and encourage smokers to quit (15).

### Raising taxes

Raising the consumer price of tobacco products is one of the most effective ways to reduce tobacco use; for every 10% increase in price, there is an estimated 4%–7% decrease in consumption (16). This effect of tobacco price increase on consumption has been found throughout the world (16,17). WHO recommends reaching or exceeding a tax rate that corresponds to at least 75% of the total cigarette price. GATS data have shown that tobacco products tend to be most affordable (measured as the ratio of tobacco price to per capita income) in countries where taxes on these products are low (e.g., cigarettes in Russia or bidis in India) (18). Higher taxes can reduce the relative affordability of tobacco products, discouraging consumption. However, the affordability of tobacco products has been on the rise in most of the world (4).

## The Years Ahead

Governments worldwide collect nearly $133 billion in tobacco excise tax revenue each year (4). Despite this, less than $1 billion is spent globally on tobacco control, with 97% of such spending occurring in high-income countries (2). In contrast, tobacco use costs the world economy an estimated $500 billion each year in health-care expenditures, productivity losses, fire damage, and other costs (19). Without effective global tobacco control efforts, low-income and middle-income countries with high population densities will continue to suffer the most harm. Even a modest decline in smoking prevalence from 25% to 20%, achieved through broader implementation of MPOWER strategies, could prevent 100 million global deaths from tobacco use by the end of the century (20).

## Figures and Tables

**FIGURE 1:**
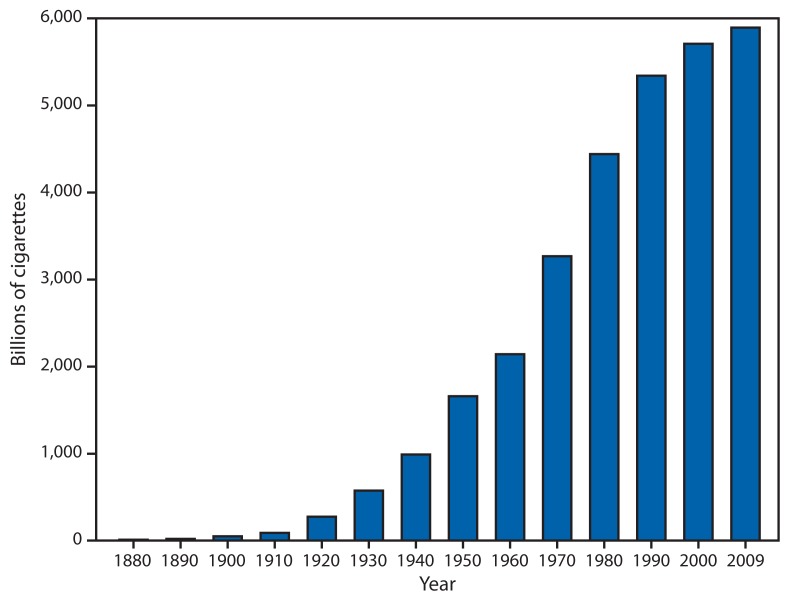
Annual global cigarette consumption — 1880–2009 **Source:** Eriksen M, Mackay J, Ross H. The tobacco atlas. Fourth ed. Atlanta, GA: American Cancer Society; New York, NY: World Lung Foundation; 2012. Available at http://www.tobaccoatlas.org.

## Footnotes

* GTSS data are available by country, WHO region, or MPOWER indicator at http://apps.nccd.cdc.gov/gtssdata/default/default.aspx.
